# Single-Stage Hybrid Arch Repair for Patients with Shaggy Aorta

**DOI:** 10.1055/s-0039-3401996

**Published:** 2020-02-12

**Authors:** Yasuhisa Oishi, Satoshi Kimura, Hiromichi Sonoda, Akira Shiose

**Affiliations:** 1 Department of Cardiovascular Surgery, Kyushu University Hospital, Fukuoka, Japan

**Keywords:** arch aneurysm, shaggy aorta, hybrid operation

## Abstract

Operating on extended arch aneurysms that contain severe atherosclerotic plaques is difficult. In such cases, the incidence of intraoperative multiple embolization is very high. We applied single-stage hybrid arch repair, which involved ascending aorta replacement and debranching of arch vessels, consecutively with endovascular repair for two patients. This technique was developed to prevent embolization of atherosclerotic plaques during cardiopulmonary bypass, and abrasion of the plaques during thoracic endovascular repair. Both patients recovered without embolic complications.

## Introduction

Thoracic endovascular aortic repair (TEVAR) is widely used for extensive arch aneurysm repair, although it has some anatomical restrictions. Further, severe atheroma is a significant risk factor for cerebral infarction during TEVAR. 1 2 To resolve these problems, we developed a new method of single-stage hybrid total arch repair.

## Case Presentation

Ethical approval was obtained from the Institutional Review Board of Kyushu University Hospital, and informed consent for surgery was obtained from the patients.

**Fig. 1:**
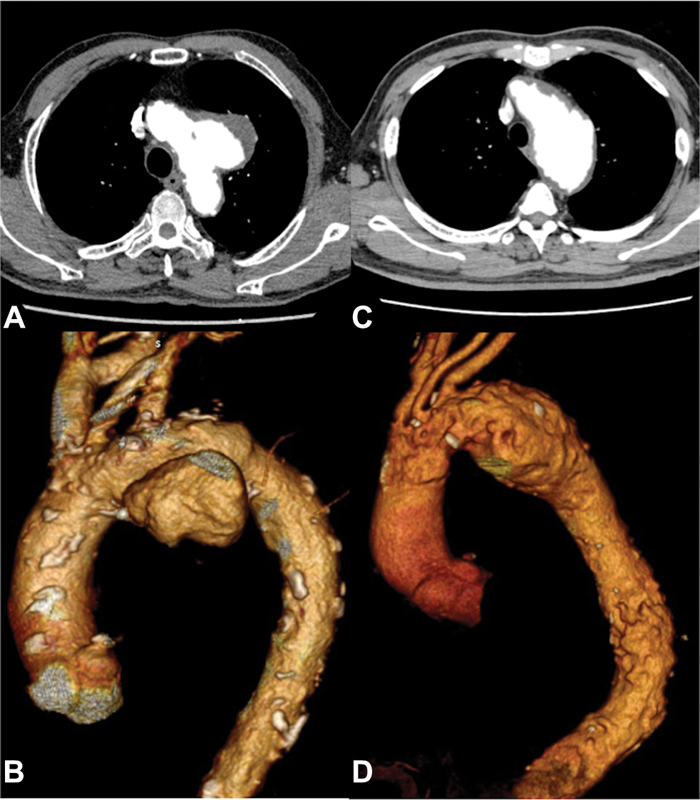
( **A, B** ) Axial and three-dimensional (3D) view of computed tomography (CT) scans in patient 1. **(C, D)** Axial and 3D view of CT scans in patient 2.

We applied our method to two patients with aortic arch aneurysms with severe atherosclerotic plaques. One patient was 80-year-old male who had a history of carotid endarterectomy. Another patient was 66-year-old male who had a history of graft replacement for abdominal aortic aneurysm. His thoracic aneurysm extended from the arch to below the pulmonary hilus. The computed tomography scans of both patients are shown in Fig. 1 .

**Fig. 2:**
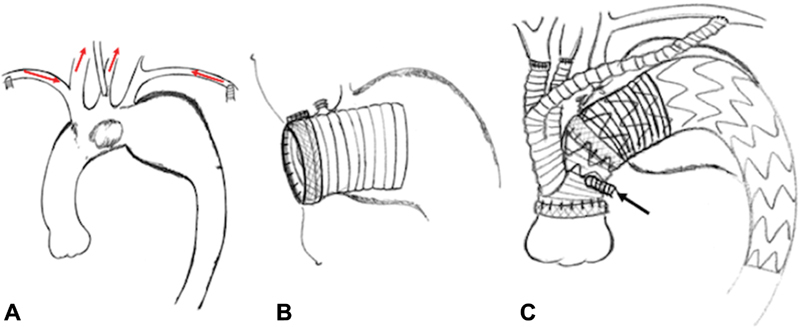
( **A** ) To avoid brain embolization caused by turbulent flow of systemic perfusion, we inserted a small perfusion cannula from the healthy side wall of left carotid artery. Bilateral axillary and left carotid arterial perfusion were started at the beginning of cardiopulmonary bypass. ( **B** ) Under selective antegrade cerebral perfusion and circulatory arrest, an elephant trunk was inserted from the distal stump. The origin of the brachiocephalic and left carotid arteries was closed. ( **C** ) After completion of all anastomosis, cardiopulmonary bypass was weaned. A stent graft was deployed antegradely from the side graft (arrows).

The patients were placed in the supine position, and a midline sternotomy was performed after completion of side-graft anastomoses to the bilateral axillary arteries. The brachiocephalic (BCA), left carotid (LCA), and left subclavian arteries (LSCA) were carefully exposed. To avoid embolization of the LCA caused by turbulent flow of systemic perfusion, we inserted a small perfusion cannula (A272–35N/J; LivaNova Inc., London, United Kingdom) through the healthy side wall. Bilateral axillary and LCA perfusion were started at the beginning of CPB ( Fig. 2A ).

After systemic cooling was established, the proximal ends of the arch vessels were clamped, and selective antegrade cerebral perfusion was initiated. The ascending aorta was transected, and cardioplegia was then initiated. The proximal ends of the BCA and LCA were closed and transected. The distal aortic stump was located between the BCA and LCA, on the proximal side of the aneurysm. A Dacron graft was carefully inserted from the distal stump as an “elephant trunk” ( Fig. 2B ) to obtain a sufficiently long landing zone and to avoid multiple embolizations caused by abrasion of mobile plaques during TEVAR. Distal and proximal anastomosis was performed using the branched Gelweave Lupiae graft (Vascutek Terumo Inc., Scotland, United Kingdom). After completion of the proximal anastomosis, systemic circulation and coronary perfusion were resumed by left axillary perfusion and the side graft of the Lupiae graft.

We reconstructed the LCA and BCA in order during systemic rewarming. The LSCA was reconstructed extra-anatomically using the already anastomosed graft to the left axillary artery. The proximal side of the LSCA was ligated to prevent Type-II endoleak.

After CPB weaning, antegrade TEVAR was performed from the side graft as an access route ( Fig. 2C ).

The postoperative courses of both patients were uneventful. They were discharged to their homes without neurological complication on postoperative days 25 and 14, respectively.

## Discussion

The “isolation technique” was previously developed to prevent brain embolisms derived from atheromatous plaques during CPB. 3 In the present study, we report our modification of this technique of selective perfusion at the initiation of CPB, in which we used selective cannulation to avoid brain embolism caused by turbulent flow during CPB. Kent et al 4 previously reported the Type-II hybrid arch repair technique for extended arch aneurysms. However, even when using that technique, we find it impossible to prevent abrasion of the atheromatous plaques during TEVAR, as the plaques in the arch remain uncovered. Thus, we modified the technique by applying an elephant trunk to cover the plaques. We did not use retrograde TEVAR in our cases, as this can increase the risk of multiple embolization. Indeed, cerebral embolization was reported following stent graft deployment, as well as contrast injection. 2 Thus, retrograde insertion of devices should be avoided if possible. Proximal arch landing is also a significant risk factor of cerebral embolism during TEVAR. 2 Placement of an endograft in a segment of replaced Dacron aorta may reduce the risk of stroke, as well as reduce endoleak, because of mechanical stability and decreased remodeling of the landing zone. 5 We have some experience of two-stage hybrid repair for arch and descending aortic aneurysms. In comparison with two-stage procedure, our single-stage method is useful in treatment of extended arch aneurysms with severe atherosclerotic plaques.
